# Characterization of *agr* Groups of *Staphylococcus pseudintermedius* Isolates from Dogs in Texas

**DOI:** 10.1128/mSphere.00033-19

**Published:** 2019-03-27

**Authors:** Sara V. Little, Laura K. Bryan, Andrew E. Hillhouse, Noah D. Cohen, Sara D. Lawhon

**Affiliations:** a Department of Veterinary Pathobiology, College of Veterinary Medicine & Biomedical Sciences, Texas A&M University, College Station, Texas, USA; b Texas A&M Institute for Genome Sciences and Society, College of Veterinary Medicine & Biomedical Sciences, Texas A&M University, College Station, Texas, USA; c Department of Large Animal Clinical Sciences, College of Veterinary Medicine & Biomedical Sciences, Texas A&M University, College Station, Texas, USA; University of Kentucky

**Keywords:** MRSP, *Staphylococcus pseudintermedius*, United States, *agr* group, *agrD*, autoinducing peptide, biofilm, clonal complex, methicillin resistance, multidrug resistance, quorum sensing, virulence factors

## Abstract

Staphylococcus pseudintermedius is an important canine pathogen implicated in an increasing number of human infections. Along with rising levels of methicillin and multidrug resistance, staphylococcal biofilms are a complicating factor for treatment and contribute to device, implant, and surgical infections. Staphylococcal virulence, including biofilm formation, is regulated in part by the quorum sensing accessory gene regulator system (*agr*). The signal molecule for *agr*, known as the autoinducing peptide molecule, contains polymorphisms that result in the formation of distinct groups. In S. pseudintermedius, 4 groups (i.e., groups I, II, III, and IV) have been identified but not comprehensively examined for associations with infection type, virulence factor carriage, or phylogenetic relationships—all of which have been found to be significant in S. aureus. In this study, 160 clinical canine isolates from Texas, including isolates from healthy dogs (*n* = 40) and 3 different infection groups (pyoderma, urinary tract, and surgical, *n* = 40 each), were sequenced. The *agr* group, biofilm-producing capabilities, toxin gene carriage, antimicrobial resistance, and sequence type (ST) were identified for all isolates. While no significant associations were discovered among the clinical infection types and *agr* groups, *agr* II isolates were significantly less common than any other group in diseased dogs. Furthermore, *agr* II isolates were less likely than other *agr* groups to be multidrug resistant and to carry toxin genes *expA* and *sec-canine*. Fifty-two (33%) of the 160 isolates were methicillin resistant, and the main sequence types (ST64, ST68, ST71, ST84, ST150, and ST155) of methicillin-resistant strains of S. pseudintermedius (MRSP) were identified for the geographic region.

**IMPORTANCE** Staphylococcus pseudintermedius is an important disease-causing bacterium in dogs and is recognized as a growing threat to human health. Due to increasing multidrug resistance, discovery of alternative methods for treatment of these infections is vital. Interference with one target for alternative treatment, the quorum sensing system *agr*, has demonstrated clinical improvement of infections in S. aureus animal models. In this study, we sequenced and characterized 160 clinical S. pseudintermedius isolates and their *agr* systems in order to increase understanding of the epidemiology of the *agr* group and clarify its associations with types of infection and antimicrobial resistance. We found that isolates with *agr* type II were significantly less common than other *agr* types in diseased dogs. This provides valuable information to veterinary clinical microbiologists and clinicians, especially as less research has been performed on infection associations of *agr* and its therapeutic potential in S. pseudintermedius than in S. aureus.

## Abstract (precis)

Staphylococcus pseudintermedius is an important disease-causing bacterium in dogs and is recognized as a growing threat to human health. Due to increasing multidrug resistance, discovery of alternative methods for treatment of these infections is vital. Interference with one target for alternative treatment, the quorum sensing system *agr*, has demonstrated clinical improvement of infections in S. aureus animal models. In this study, we sequenced and characterized 160 clinical S. pseudintermedius isolates and their *agr* systems in order to increase understanding of the epidemiology of the *agr* group and clarify its associations with types of infection and antimicrobial resistance. We found that isolates with *agr* type II were significantly less common than other *agr* types in diseased dogs. This provides valuable information to veterinary clinical microbiologists and clinicians, especially as less research has been performed on infection associations of *agr* and its therapeutic potential in S. pseudintermedius than in S. aureus.

## INTRODUCTION

Staphylococcus pseudintermedius is a canine commensal and opportunistic pathogen responsible for a wide range of infections, including pyoderma, bacteremia, and implant device-related and postsurgical infections. While infections of humans by S. pseudintermedius are less common than infections of dogs, identification of S. pseudintermedius as a human pathogen is being increasing reported (1–6). Growing multidrug resistance (MDR) and the spread of methicillin-resistant strains of S. pseudintermedius (MRSP) complicate treatment of these infections. Without the use of matrix-assisted laser desorption ionization–time of flight mass spectrometry (MALDI-TOF MS), it can be difficult to differentiate between S. pseudintermedius and S. aureus. Accurate identification is key because the cefoxitin surrogate for methicillin resistance testing in S. aureus often fails to detect methicillin resistance in S. pseudintermedius (5, 7, 8). Misidentification of S. pseudintermedius can therefore complicate treatment of human infections.

A major staphylococcal virulence factor is the ability to form biofilms, facilitating adherence to biotic and abiotic surfaces. *Staphylococcus* biofilms are complex in both regulation and composition. Polysaccharide intercellular adhesin (PIA), also known as slime, is among the major extracellular polymeric substances found in staphylococcal biofilms that facilitate adherence to host cells and is involved with evasion of the host’s innate immune response. Slime production can be determined by the Congo red assay; however, staphylococci can also produce protein-based biofilms that are PIA independent. Consequently, determining overall biofilm production requires the use of other methods such as crystal violet microtiter plate detection assays. Bacterial cells embedded in biofilms benefit from enhanced antimicrobial resistance (9, 10). The high density of bacteria in biofilms provides an opportune environment for density-dependent bacterial communication using signal molecules, known as quorum sensing (QS). A main virulence-regulating QS system in staphylococci is the accessory gene regulator (*agr*) system, influencing over 70 genes (11) and consisting of 4 component proteins: AgrA, AgrB, AgrC, and AgrD. The AgrD propeptide is the precursor of the *agr* QS signal known as the autoinducing peptide (AIP). After processing and export by AgrB, the AIP molecule is detected by the two-component receptor kinase AgrC/AgrA. AgrA activation leads to positive feedback of the *agr* system as well as an upregulation of proteases and toxins and a downregulation of surface proteins (11, 12). Four AIP variants (RIPTSTGFF [variant I], RIPISTGFF [variant II], KIPTSTGFF [variant III], and KYPTSTGFF [variant IV]) are unique to S. pseudintermedius (13, 14). In S. aureus, *agr* groups are associated with certain types of infections, including endocarditis, bacteremia, and toxic shock syndrome (15–18); similar associations have not been well clarified for S. pseudintermedius.

The genetic lineage of an S. pseudintermedius isolate can be determined using multilocus sequence typing (MLST). MRSP lineages are clonal and associated with specific geographical distribution and antimicrobial resistance profiles, with genetic diversity achieved through the acquisition of mobile genetic elements (19). Associations have been found between sequence types (ST) and infection sources along with a variety of virulence factors (20). Methicillin-susceptible S. pseudintermedius (MSSP) isolates lack clonality and are poorly characterized despite comprising the majority of clinical isolates.

In this study, whole-genome sequencing (WGS) was performed on 160 canine clinical isolates of S. pseudintermedius collected at the Texas Veterinary Medical Teaching Hospital (VMTH) at Texas A&M University between 2007 and 2016 in order to determine the associations between virulence characteristics, infection type, MLST, and *agr* group. Congo red assays (CRA) and crystal violet microtiter plate assays (CVA) were performed on the sequenced isolates and on an additional 550 canine clinical isolates collected during the same time period to characterize biofilm-forming capabilities.

## RESULTS

### Prevalence and distribution of *agr* groups.

All but 2 of the sequenced 160 S. pseudintermedius isolates had 100% identity to 1 of the 4 known *agrD* alleles. One exception contained a silent mutation in the AIP region, and the other contained a missense mutation in the C-terminal region resulting in a lysine instead of glutamic acid. As neither of these mutations impacted the final AIP structure, they were categorized accordingly. Eleven (7%) isolates were classified in *agr* group I, 50 (31%) in *agr* group II, 44 (28%) in *agr* group III, and 55 (34%) in *agr* group IV.

### Biofilm production.

Biofilm assays were performed on clinical isolates (*n* = 710) from individual dogs (Table 1). Slime production, determined by CRA, was found in about half of the isolates (51%; 362/710). The proportion of slime-producing isolates from diseased dogs (55%; 291/528) was significantly (*P* = 0.0002) greater than that of isolates from healthy dogs (39%; 71/182).

**TABLE 1:** Biofilm assay results^*a*^

Isolate collection	No. (%) of isolates					
	CRA negative— non-slime producing	CRA positive— slime producing	CVA 0— nonadherent	CVA 1— weakly adherent	CVA 2— moderately adherent	CVA 4— strongly adherent
Total (*n* = 710)						
Total	348 (49)	362 (51)	83 (12)	240 (34)	313 (44)	74 (10)
Healthy	111 (32)	71 (20)	9 (11)	53 (22)	98 (31)	22 (30)
Diseased	237 (68)	291 (80)	74 (89)	187 (78)	215 (69)	52 (70)
CRA negative			37 (45)	118 (49)	154 (49)	39 (53)
CRA positive			46 (55)	122 (51)	159 (51)	35 (47)

Sequenced (WGS, *n* = 160)						
Total	77 (48)	83 (52)	17 (11)	58 (36)	72 (45)	13 (8)
Healthy	23 (30)	17 (20)	2 (12)	15 (26)	21 (29)	2 (15)
Pyoderma	19 (25)	21 (25)	3 (18)	12 (21)	18 (25)	7 (54)
Urinary tract	15 (19)	25 (30)	4 (24)	18 (31)	14 (19)	4 (31)
Surgical	20 (26)	20 (24)	8 (47)	13 (22)	19 (26)	0 (0)
CRA negative			10 (59)	28 (48)	33 (46)	6 (46)
CRA positive			7 (41)	30 (52)	39 (54)	7 (54)
MRSP	16 (21)	36 (43)	9 (53)	15 (26)	23 (32)	5 (38)
MSSP	61 (79)	47 (57)	8 (47)	43 (74)	49 (68)	8 (62)

a Percentages have been rounded and may not total 100%.

The CVA optical density for the control (OD_control_) was 0.3317 (±0.1818). Overall biofilm production, measured by CVA, showed that a majority of isolates (78%; 553/710) produced weak or moderately weak biofilms. There was no significant difference in the distribution of overall biofilm production between slime-producing and non-slime-producing isolates. Overall levels of biofilm production differed significantly between healthy and diseased-sourced isolates (*P* = 0.0014), which was largely attributable to the presence of a high proportion of nonadherent isolates in the diseased category.

**FIG 1:**
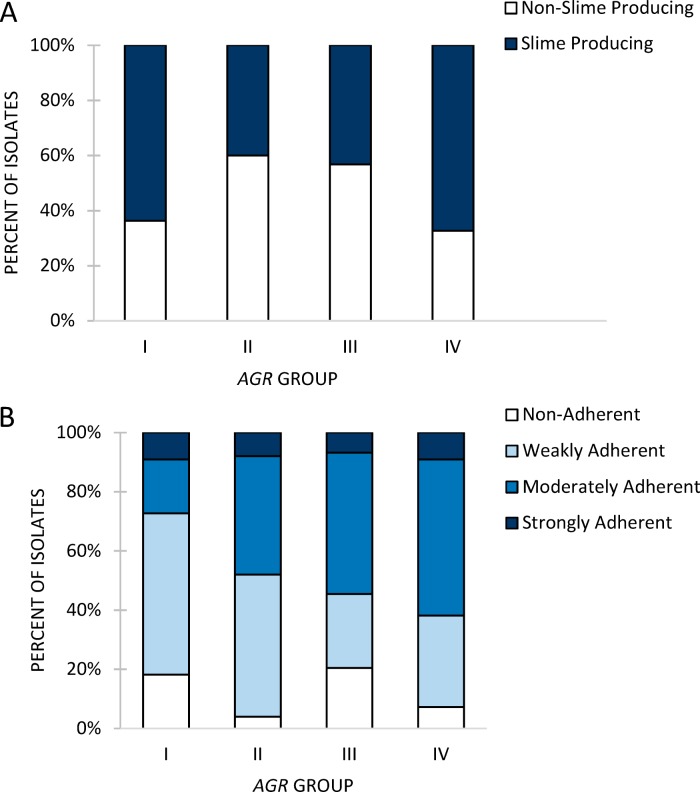
(A) Slime production as measured by CRA of *agr* groups. Slime production was significantly less associated with the *agr* II and III groups than with the *agr* I and IV groups. (B) Overall biofilm production as measured by CVA of *agr* groups.

Biofilm results for the 160 WGS isolates are given in Table 1. Approximately half of the isolates were slime producers, and there was no significant association of slime production with the disease groups as measured by CRA. With respect to overall biofilm formation, a majority of the sequenced isolates (89%; 143/160) formed biofilms, and most of those isolates (91%, 130/143) were classified as weak to moderately weak biofilm producers. No significant associations were seen between the overall levels of biofilm production and disease group or slime production status. There were no significant differences between the overall levels of biofilm production in the 4 *agr* groups (Fig. 1B). Slime production was significantly less associated with *agr* II and III than with *agr* I and IV (*P* = 0.0415) (Fig. 1A).

### Disease associations of *agr* groups.

**FIG 2:**
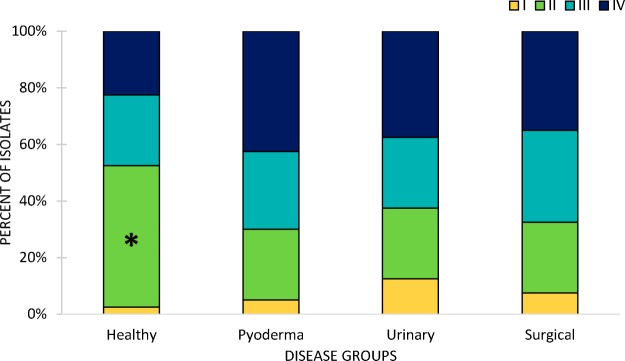
Distribution of *agr* groups across the four examined disease groups. Significance is indicated by an asterisk (*).

The data corresponding to the number of isolates from each *agr* group found in each disease group are summarized in Fig. 2. The distributions of *agr* groups differed significantly between healthy and diseased dogs (*P* = 0.025). Group II isolates were significantly more common in healthy dogs than in diseased dogs (*P* = 0.0058). No further significant associations were seen between *agr* groups and disease and the disease groups (healthy, pyoderma, urinary tract infection, and surgical infection).

### Resistance gene carriage of *agr* groups.

The majority of WGS isolates were methicillin susceptible (68%; 108/160) as determined by the lack of *mecA*. Methicillin resistance was identified for 45% (*n* = 5) of *agr* group I isolates, 18% (*n* = 9) of group II isolates, 34% (*n* = 15) of group III isolates, and 42% (*n* = 23) of group IV isolates (Table 2). The group II isolates were significantly (*P* = 0.0025) less likely to be MRSP. Isolates from healthy dogs were significantly (*P* = 0.0002) less likely to carry *mecA* than the diseased isolates. The *mecA* cassette was found in 8% of healthy isolates (*n* = 3), 48% of pyoderma isolates (*n* = 19), 30% of urinary isolates (*n* = 12), and 45% of surgical isolates (*n* = 18).

**TABLE 2:** Virulence factor carriage^*a*^

Virulence factor	% prevalence			
	*agr* group I (*n* = 11)	*agr* group II (*n* = 50)	*agr* group III (*n* = 44)	*agr* group IV (*n* = 55)
Toxin gene carriage				
*expA*	0	8	9	22**
*expB*	27	20	7*	4*
*sec-canine*	0*	2*	14	16
*speta*	100	100	100	100
*siet*	100	100	100	100
*luk-S/F*	100	100	100	100
*se-int*	100	100	100	100

Antimicrobial resistance and gene carriage				
*mecA*	45	18*	34	42
Aminoglycoside	45	34	32	51
Beta-lactam	82	84	82	95
Chloramphenicol	0	4	9	4
MLS	45	18*	34	45
Tetracycline	55	36*	61	69
Trimethoprim	9*	12*	32	44
MDR	45	16*	34	47

a A single asterisk (*) signifies that the members of the indicated *agr* group are significantly less likely to be carried; double asterisks (**) signify that the members of the indicated *agr* group are significantly more likely to be carried.

Data representing the carriage of genes for resistance to selected antimicrobials are presented in Table 2. Sixty-four isolates carried genes for aminoglycoside resistances [*aac(6')-aph(2'')*, *ant(* 6*)-la*, or *aph(3′)-III*], but there was no significant association between *agr* types and aminoglycoside resistance. There was a significant association between the *agr* groups and macrolide, lincosamide, and streptogramin (MLS) resistance gene carriage [*erm(B)* and *inu(A*)], with the proportion of MLS gene carriage significantly (*P* = 0.0171) lower in *agr* group II isolates. Similarly, the proportion of tetracycline resistance carriage [*tet*(K) and *tet*(M)] was significantly (*P* = 0.0057) lower in group II. For trimethoprim resistance, there was a significant association between *agr* types and resistance; the proportion of isolates carrying the *dfrG* resistance gene among *agr* group I or II isolates was significantly (*P* = 0.0005) lower than that seen with isolates of group III or IV. There was no association seen between *agr* group and beta-lactam resistance gene carriage, as most isolates (*n* = 139) carried *blaZ*. No association was seen between *agr* group and chloramphenicol resistance, as most isolates (*n* = 152) did not carry *cat*(*pC221*).

There was a significant association between *agr* group and MDR as defined by resistance to 3 of the 5 classes of antibiotics other than beta-lactams. Group II isolates were significantly (*P* = 0.0045) less likely to be MDR. Only 16 isolates, consisting of a mix of *agr* types, were susceptible to all drug classes.

### Toxin carriage of *agr* groups.

There was universal carriage of the *speta*, *siet*, *luk-S/F*, and *se-int* toxin genes. Twenty isolates carried *expA*, and 17 carried *expB*, with 1 isolate carrying both genes. *agr* group IV isolates were significantly (*P* = 0.0199, Table 2) more likely to carry *expA* than the isolates in the other *agr* groups were. The overall distributions of *agr* groups also differed significantly by *expB* carriage, as isolates of *agr* groups III and IV were significantly (*P* = 0.0037, Table 2) less likely to carry *expB*. Sixteen isolates carried *sec-canine*, with *agr* groups III and IV significantly (*P* = 0.0126) more likely to carry *sec-canine* than groups I and II. Finally, one isolate carried the gene for the bacteriocin-like peptide BacSp222 (21).

### Phylogenetic relationships and clonal complexes based on MLST.

MLST determined 91 new allele combinations, 31 new alleles, and 113 new strain types submitted to the S. pseudintermedius pubMLST database. Results of PHYLOViZ (phylogenetic inference and data visualization) analysis (22) performed in August 2018 suggested the major S. pseudintermedius clonal complexes (CC) represented in this study and in all submitted North American isolates to be CC45, CC64, CC68, CC71, CC84, CC150, and CC155. The most highly represented STs in our isolates were ST64, ST68, ST71, ST84, ST150, and ST155. Statistically significant associations were seen between ST64 and *agr* II (*P* = 0.012, 5/5 isolates were *agr* II), between ST68 and *agr* IV (*P* < 0.0001, 13/13), between ST71 and *agr* III (*P* = 0.0005, 7/7), and between ST 84 and *agr* I (*P* < 0.0001, 5/5); ST150 and ST155 had too few isolates (*n* = 3 each) for assessment of statistical significance, but all members were *agr* group IV.

The proportion of isolates outside these main STs was significantly lower in diseased dogs than in healthy dogs (*P* = 0.0162). ST71 isolates were significantly more likely to be derived from surgical cases (*P* = 0.0111). STs also differed significantly by slime production (*P* = 0.0014); however, there was no evidence that individual STs were significantly associated with slime production. The distributions of overall biofilm production differed significantly (*P* = 0.0032) between ST71 and the other types by CVA, with a higher proportion of ST71 isolates being nonadherent.

Overall, MRSP isolates were more likely to be MDR (*P* < 0.001), and ST associated with methicillin resistance were significantly (*P* < 0.0001) associated with MDR; ST64, ST68, ST71, ST84, ST150, and ST155 were more likely to be MDR than other STs and significantly (*P* < 0.0001) more likely to carry *mecA*. These STs were also more likely to carry genes conferring resistance to aminoglycosides (*P* < 0.0001), beta-lactams (*P* = 0.0043), MLS (*P* < 0.0001), tetracycline (*P* = 0.0003), and trimethoprim (*P* < 0.0001).

For toxin carriage, there was a significant association of ST with *expA* carriage (*P* = 0.0142); ST155 isolates were significantly (*P* = 0.0017) more likely to be *expA* positive. No association was seen with *expB* carriage, but ST155 and ST150 were significantly (*P* = 0.0017) more likely to carry *sec-canine*.

## DISCUSSION

The majority of the 710 clinical isolates (90%) were biofilm producers, which agrees with the current literature on biofilm production in *Staphylococcus* clinical isolates (23, 24). Slime production was significantly more common in isolates from diseased dogs but was seen in only half of isolates, which is a lower rate than had been previously reported (25). All but 4 WGS isolates (98%; 156/160) contained the required *icaA* and *icaD* genes for PIA (slime) production, which suggests that, similarly to observations in S. aureus, isolates may have the *ica* locus but may still fail to produce slime *in vitro* due to sensitive growth conditions (25–27). These results highlight the importance of a combination of phenotypic and genotypic methods for examining biofilm formation in S. pseudintermedius.

The global population structure of S. pseudintermedius has been well examined. A 2016 review of 58 studies determined the following 5 major MRSP CCs worldwide: CC71, CC258, CC68, C45, and CC112 (19). However, PHYLOViZ analysis at that time assigned the isolates into only 2 major (CC71 and CC258) and 2 minor (CC379 and CC75) clonal complexes (19). Our PHYLOViZ analysis of the 1,047 isolates present in the S. pseudintermedius PubMLST database at the time of this study, including our 160 isolates, identified only 1 major CC (CC258) comprising all of the major STs. This suggests that MLST lacks the power to separate the groups, a problem recently observed in S. aureus (28).

Worldwide, ST71 is the most predominant MRSP lineage, followed by ST45, ST258, ST261, ST112, ST265, ST68, ST169, and ST181 (19). A 2018 study of 190 North American dog isolates determined the region’s major STs to be ST68, ST71, and ST84 (29). While our study also identified ST68, ST71, and ST84 as major North American STs, we discovered ST64 to be similarly highly represented. Unpublished data that include an additional 32 sequenced isolates suggest that seven major North American STs, namely, ST45 (*n* = 6), ST64 (*n* = 5), ST68 (*n* = 13), ST71 (*n* = 7), ST84 (*n* = 5), ST150 (*n* = 3), and ST155 (*n* = 3), are represented among our clinical isolates, which represents the first time that ST45 has been found to be a major ST in the United States. It should be noted that this finding is not representative of North America as a whole, as sample collection was limited to dogs in Texas.

MRSP STs are known to be associated with virulence factor carriage, MDR, and *agrD* alleles; indeed, in the previous 5-gene MLST scheme, *agrD* was among the genes used to determine ST (14). Each of the major STs identified in this study is associated with a specific *agr* group, highlighting a lack of assortative recombination within these specific STs in our collection. While this represents a small sample set from which to draw conclusions, the data may suggest that, similarly to S. aureus, the evolutionary divergence of the *agr* locus into separate groups occurred prior to clonal diversification and that recombination played a lesser role in *agr* distribution (30).

Biofilm associations are more poorly understood; previously, ST71 was identified as the strongest biofilm former of any MRSP lineage (31). Here, ST71’s distribution of CVA scores differed significantly from the distributions seen with the other types due to a slight majority of ST71 isolates being nonadherent whereas the rest were moderately adherent, in contrast to previous studies. Interestingly, ST71 was associated with surgical infections, where biofilm and slime production may be important, again supporting the idea of the complex conditions required for *ica* activation, with variation between isolates (32). The main ST groups were also all more likely to be MDR, highlighting their clinical importance.

The majority of our clinical isolates belonged to new, unique STs that are less closely related to the main 6 MRSP STs per PHYLOViZ analyses. These were associated with colonization of healthy dogs and were less likely to be MDR. Most were MSSP, which is consistent with the known lack of clonality among MSSP isolates (29, 33). MSSP isolates represent a less extensively studied but still clinically relevant group that comprises the majority of S. pseudintermedius isolates. Due to their lack of clonality and unique STs, associations between MSSP STs and virulence factors are difficult to determine, but associations between *agr* group and infection and virulence profiles can still be examined.

The prevalence of *agr* groups in our clinical collection is similar to that seen previously (14), with *agr* I being the least prevalent group whereas *agr* II, III, and IV were similarly distributed at approximately 30%. Within our collection, the members of *agr* group II were more commonly associated with commensal carriage and methicillin susceptibility and less likely to be slime producers or MDR or to carry either *expA* or *sec-canine* while being more likely to carry *expB*. In contrast, the members of *agr* groups I and IV were more likely to be slime producers and MDR. Previously, *agr* group III was determined to be more strongly associated with MRSP than the other *agr* groups (34), but this was not observed for our isolates. The lack of association with pyoderma or surgical or urinary tract infections is not necessarily indicative of a lack of association of *agr* group and infections; such associations may be based on the severity of infection or on infection types that we were unable to examine in this study.

We found that healthy dogs were less likely to carry MRSP isolates than diseased dogs, but this result was likely due, in part, to selection bias in the group of diseased dogs. Our isolates were cultured from patients presented to a teaching hospital that includes both primary and tertiary care services. Most isolates came from patients presented to referral services and were likely cultured due to the presence of an infection that had not responded to empirical antimicrobial treatment. The surgical disease group was cultured at first appearance of problems without any prior treatments, but the urinary tract disease group and pyoderma disease group were potentially subject to the referral hospital bias. Healthy dogs were candidates for orthopedic surgery; while not entirely healthy, they had no visible skin lesions and no recent history of antimicrobial therapy. One limitation to our study was that we sequenced only 40 healthy isolates and 120 diseased isolates. This impacted statistical power and may explain the lack of difference between the levels of biofilm and slime production detected in the diseased and healthy groups. We attempted to limit selection bias by randomly selecting isolates in the healthy, urinary tract infection, and pyoderma groups for sequencing. All available isolates from surgical site infections were sequenced.

Understanding the clinical associations of *agr* groups is becoming increasingly important as demands for alternative methods of treatment grow due to antimicrobial resistance. The *agr* system is an alternative target for therapeutics in S. aureus, where inhibition of the *agr* system has demonstrated effects on virulence *in vivo*, including increased survival, blocked abscess formation, decreased lesion size, hindered progression of skin infections, and improved host defense in murine models (30, 35–38). The judicious use of antimicrobials in veterinary practice represents an attempt to limit the spread of resistance to classes of drug considered important or of last resort in human medicine. For this reason, vancomycin is not used in small animals at the veterinary hospital where these isolates were collected. As MRSP strains resistant to all drugs used for canine patients are frequently encountered in our patient population, there is a strong need for alternative therapies to treat resistant and pervasive infections. Disruption or interference with the quorum sensing system of S. pseudintermedius to reduce virulence represents a potential avenue of treatment that would not result in application of selective pressures for antibiotic resistance. Furthermore, *agrD* alleles are known to act within and between species as potent *agr* inhibitors, and those of S. pseudintermedius, while not comprehensively examined, have demonstrated effects against S. aureus (35). Examining the cross-interference potential of the four S. pseudintermedius AIPs will be important, not only for evaluation of its utility as a natural reservoir for potential therapeutics but also to understand the effects of cocolonization and potential clinical implications for patients colonized by S. pseudintermedius alongside S. aureus or S. epidermidis.

## MATERIALS AND METHODS

### Bacterial isolates and demographics.

Staphylococcus pseudintermedius strains collected between 2007 and 2016 were acquired from the Clinical Microbiology Laboratory (CML) of the VMTH. The isolates were presumptively identified at initial collection as S. pseudintermedius based on the standard operating procedure of the laboratory, which included Gram stain, colony color, polymyxin B susceptibility, production of coagulase and catalase, and ability to grow on salt-mannitol agar prior to June 2016 and included identification to the level of the Staphylococcus intermedius group via matrix-assisted laser desorption ionization–time of flight mass spectrometry (MALDI-TOF MS) after June 2016. Species identification was performed for all isolates in the CML using PCR as previously described (39). Isolates were collected from dogs and stored in glycerol lysogeny broth (LB) at −80°C at the time of isolation. The group of isolates representing disease (*n* = 528) were residual patient diagnostic samples that had been collected from dogs with various disease conditions and that had been isolated from different culture sources, among which skin (*n* = 319) and urine (*n* = 81) were the most common. The isolates from dogs with healthy skin (healthy isolates; *n* = 182) were collected for a staphylococcal screening study that cultured samples from the nares and perineal skin of dermatologically healthy dogs being evaluated for elective orthopedic surgery. All sampling conformed to the ethical guidelines and standards of care for the hospital. The Institutional Animal Care and Use Committee and Clinical Research Review Committee approved the collection of isolates from the healthy dog group (AUP 2010-068).

For this study, all cultures were grown on Trypticase soy agar (TSA) with 5% sheep blood (blood agar; BBL, USA) and incubated at 37°C for 24 h. ATCC S. epidermidis strain 12228 and ATCC S. aureus strain 25923 were used as negative and positive controls, respectively, for biofilm production assays.

For sequencing, 40 isolates were randomly selected from each of the following source groups: healthy dogs and dogs with pyoderma, urinary tract infection, and surgical infection (*n* = 160). The number of isolates was limited to 40 due to the availability of isolates from surgical site infections in the collection. Surgical infections were defined as cases where no clinical infections existed preoperatively and included infections of bone and implant hardware and incision line culture sites.

### Phenotypic characterization of slime production on Congo red assay (CRA).

To assess the ability of isolates to produce slime as characterized by PIA production, isolates were cultured on Congo red agar plates. Plates were prepared and incubated as previously described (40). Slime production was determined by the color and texture of the colony surface and was scored on the basis of a 5-point scale system (adjusted from a method described previously by Arciola et al. [40]) as follows: 0, smooth, red; 1, smooth, moderately red; 2, smooth, dark red (Bordeaux) (the isolates with a score of 0, 1, or 2 were designated non-slime-producing isolates); 3, rough, almost black; 4, rough, completely black (the isolates with a score of 3 or 4 were designated slime-producing isolates). Plates were grown in biological and technical duplicate.

### Phenotypical characterization of biofilm production on crystal violet microtiter plate assay (CVA).

Overall biofilm production was assessed by the ability of the isolates to adhere to a 96-well microtiter plate as previously described (25, 41). The CVA was performed based on the published protocols, with the exception that cultures were diluted 1:200 in Trypticase soy broth (TSB)–1% glucose to encourage biofilm production in flat-bottomed, tissue culture-treated polystyrene microtiter plates (Nunc-Immuno, USA), and the resultant rinsed and dried biofilms were stained with 0.1% crystal violet dye. The values corresponding to the optical density at 570 nm (OD_570_) were adjusted for each plate by subtracting the average of the values determined for the blank control wells (TSB broth only). Cutoff values for biofilm production were established using a previously published 4-point scale (25), where 0 represents nonadherence (OD ≤ OD_control_), 1 represents weak adherence (OD_control_ < OD ≤ 2 × OD_control_), 2 represents moderate adherence (2 × OD_control_ < OD ≤ 4 × OD_control_), and 3 represents strong adherence (4 × OD_control_ < OD). Microtiter plate assays were performed in technical and biological triplicate.

### DNA extraction and library preparation.

Isolates from source freezer stocks were grown on blood agar for 24 h at 37°C and subcultured twice to ensure the purity of the cultures. A single colony was inoculated into brain heart infusion broth (BD, USA) and grown for 8 h. A 1-ml aliquot was used for DNA extraction via the use of a MasterPure Gram-positive DNA purification kit, per their standard protocol. DNA was quantified using the Life Technologies Qubit high-sensitivity (HS) double-stranded DNA (dsDNA) assay, and all samples were normalized to 100 ng of DNA in a total volume of 14 μl. Sequencing libraries for 2 separate Illumina MiSeq sequencing runs were prepared by hand using a BiOO Scientific NEXTflex rapid DNA-Seq kit per their standard protocol. Prepared libraries were checked with the Qubit high-sensitivity dsDNA assay to determine concentrations and with an Agilent TapeStation D1000 HS system to determine the average fragment size for the prepared libraries. All samples were normalized to 4 nM and pooled per run. The 4 nM pool was diluted to 20 pM and sequenced on the Illumina MiSeq 300×300-cycle v3 sequencing kit. This resulted in approximately 500,000 paired-end reads per isolate and in approximately 50× coverage. All run data and FASTQ files were uploaded to BaseSpace (Illumina) for downstream analysis.

### Genome assembly and alignment.

The Illumina MiSeq reads were assembled using SPAdes *de novo* genome assembly (version 3.6.2) on the Texas A&M Institute for Genome Sciences and Society (TIGSS) computing cluster, with the following parameter: –careful. The resulting assemblies were annotated using the RAST tool kit (RASTtk) at the Pathosystems Resource Integration Center (PATRIC) (42).

### *agr* typing and virulence gene carriage.

In order to determine the *agr* group, PATRIC’s BLASTn function was used on the assembled genomes using previously published sequences for the 4 *agrD* alleles (GenBank accession no. EU157356.1, EU157391.1, EU157400.1, and EU157402.1) (14). BLAST was similarly used to identify toxin carriage of known S. pseudintermedius toxin genes (*speta*, *siet*, *expA*, *expB*, *Luk-S/F*, *sec-canine*, *se-int*, *BacSp222*), and ResFinder 2.1 (43) was used to check for antimicrobial gene carriage for the following antimicrobial resistance gene categories: aminoglycoside; beta-lactam; colistin; fluoroquinolone; fosfomycin; fusidic acid; glycopeptide; macrolide, lincosamide, and streptogramin (MLS); nitroimidazole; oxazolidinone; phenicol; rifampin; sulfonamide; tetracycline; and trimethoprim.

### MLST typing.

Multilocus sequence typing was performed using the 7-gene MLST scheme utilized by the Staphylococcus pseudintermedius MLST database (https://pubmlst.org/spseudintermedius/; curator, Vincent Perreten) (44). The genes used for MLST included *ack*, *cpn60*, *fdh*, *pta*, *purA*, *sar*, and *tuf*. FASTA sequences were submitted to the MLST database, and then MLST was determined based on the bacterial isolate genome sequence database (BIGSdb) (44). Potential new alleles were identified via BLASTP, and the FASTA sequences were submitted to the database for allelic determination and final ST assignment. Clonal complexes and the phylogenetic relationships of these isolates were examined using the default settings for PHYLOViZ analysis (22), which utilizes the goeBURST algorithm, a refinement of the eBURST algorithm (45). CC classifications were determined using a previously published method where STs share at least 6 identical alleles and the primary founders have the greatest number of single-locus variants (19).

### Statistical analysis.

Statistical significance was determined using the Chi-square test or, when the value in any cell exceeded the expected value by <5, Fisher’s exact test. Data were analyzed using contingency tables (*k* × *k*, as indicated for the specific comparison; some tables were 2-by-2 layouts, whereas others exceeded dichotomous levels). Significance for analysis was set at *P* values of <0.05.

### Data availability.

The 160 WGS annotated genomes and contigs are available to the public on PATRIC (https://patricbrc.org) and can be found in the PATRIC public workspaces portal under the name SLittle_StaphPseudAGR. The accession numbers are 283734.1312 to 283734.1471.
